# Health Literacy Directed Weight Loss Intervention in Primary Care Clinics

**DOI:** 10.3928/24748307-20240618-01

**Published:** 2024-10

**Authors:** Terry Davis, Connie Arnold, Dachuan Zhang, Corby K. Martin, Robert L. Newton, Candice Myers, Kara D. Denstel, Emily F. Mire, Christoph Höchsmann, John Apolzan, Peter T. Katzmarzyk, for the PROPEL Research Group

## Abstract

**Background:**

Low income and low health literacy are associated with poorer health knowledge, health behaviors and poor health outcomes. The effectiveness of health literacy-directed weight loss treatment interventions in primary care clinics is lacking.

**Objective:**

The aim of this study was to conduct a pragmatic cluster-randomized trial (PROmoting Successful Weight Loss in Primary CarE in Louisiana ([PROPEL]) to test the effectiveness of a 24-month, patient-centered, literacy-directed obesity treatment program delivered within primary care in an underserved population. This study reports the association between health literacy and program effectiveness, examining potential correlates of weight loss related to patient adherence to the program.

**Methods:**

We randomly assigned 18 clinics to usual care (UC) or a health literacy-directed lifestyle intervention (HLI). The primary outcome was percent weight loss at 24 months.

**Key Results:**

Of 803 adult participants (84% women; 67% Black), 31% had limited health literacy. Patients in UC lost an average of 0.44%of their enrollment weight after 24 months. Those with adequate literacy lost 0.57% and those with limited literacy lost 0.30%, which was not significantly different. The HLI patient group lost an average of 4.9% of their enrollment weight. Those with adequate literacy lost 5.2% and those with limited literacy, 4.7%, which was not significantly different. The advantage of adequate health literacy was consistent across the 24-month study period, though not significant. Patients in the HLI group with adequate health literacy had greater percent weight loss by a margin of 0.50 ± 0.75 (*p* = .50), while the UC margin was 0.27 ± 0.84 (*p* = .74). The percent weight loss difference between HLI and UC groups was 4.6 ± 0.8 (*p* < .001) among patients with adequate health literacy and 4.4 ± 1.0 (*p* < .001) among patients with limited health literacy. The difference in percent weight loss between the HLI and UC groups was 0.2 ± 1.1 (*p* = .84) higher for adequate literacy patients.

**Conclusions:**

A health literacy directed health coaching intervention in community clinics led to significant weight loss over 24 months but did not vary by level of patient health literacy. [***HLRP: Health Literacy Research and Practice*. 2024;8(4):e204–e211.**]

## Abstract (plain language summary)

**Plain Language Summary: **Our 2-year health literacy-appropriate obesity treatment program delivered in community clinics was effective in engaging underserved patients with low income and helping them lose weight. The personal health coaching strategy mitigated the impact of low health literacy. Patients who received more in-person coaching sessions lost more weight, suggesting literacy appropriate materials and communication with the health coach are important to weight loss success.

Low income and low health literacy are associated with poorer health knowledge and health behaviors and poor health outcomes, which lead to health inequities (Meherali et al., 2020; O'Conor et al., 2020; Schaffler et al., 2018). This is particularly concerning in addressing the pervasive problem of obesity and associated chronic diseases. It is estimated that 36% of adults in the United States have limited health literacy and 42% have obesity (Fryar, 2020; Hales et al., 2018; Katzmarzyk et al., 2021; U.S. Department of Health and Human Services, 2010). Rates for these problems are even higher among individuals with low income, racial and ethnic minority populations, and those living in rural areas (Katzmarzyk et al., 2021; Kutner et al., 2006; Ogden et al., 2020; Steinberg et al., 2019; Ward et al., 2019).

Individuals in these groups are more likely to experience health literacy barriers to accessing, understanding, and using health information and services to make informed health decisions (U.S. Department of Health and Human Services, 2010). Culturally appropriate interventions are needed to address these pervasive health disparities (Faruqi et al., 2015; Hales et al., 2018; Katzmarzyk et al., 2021; O'Conor et al., 2020; Steinberg et al., 2019). Primary care and community clinics that commonly serve vulnerable groups could be an ideal setting for weight loss treatment. However, many primary care providers (PCP) are reluctant to treat obesity due to lack of time and training, low reimbursement, and a belief that counseling will be ineffective (de Lannoy et al., 2021; Katzmarzyk et al., 2021). Despite concerns that rates of obesity show no signs of decreasing, the number of patients receiving weight loss counseling from PCPs has declined substantially over time (Katzmarzyk et al., 2021; Katzmarzyk et al., 2020; Kraschnewski et al., 2013).

Weight loss counseling by PCPs alone, without the support of a comprehensive weight loss intervention, is commonly ineffective (McVay et al., 2019). Higher intensity, multicomponent interventions in primary care clinics delivered by trained health coaches may have the potential to produce greater weight loss (Katzmarzyk, 2023; Semlitsch et al., 2019; Wadden et al., 2014).

Numerous options for weight loss treatment are available, such as changes in diet and physical activity, behavioral management, regular contact with a trained interventionist, pharmacological treatments, and referrals for bariatric surgery when all nonsurgical interventions have failed (de Lannoy et al., 2021; Michalopoulou et al., 2022; Semlitsch et al., 2019). A systematic review of international, evidence-based guidelines for the management of overweight and obesity in high-income countries found treatment needs to include a comprehensive lifestyle intervention by a multidisciplinary team for at least 6 to 12 months with a goal of 5% to 10% weight reduction (Semlitsch et al., 2019). Longer-term measures to maintain weight loss are also needed.

Weight loss studies that involve populations with low income and low health literacy have included regular phone calls from a dietitian, interactive voice response or text or prompts through clinic portals, and digital interventions (Lanpher et al., 2016; McVay et al., 2019; Steinberg et al., 2019). A Swiss review of literature on the effects of health literacy interventions designed for socioeconomically disadvantaged adults found culturally appropriate patient-centered interventions that used tailoring, skill training, goal setting, and active discussion with an intervention provider over multiple sessions were needed to aid patients with limited literacy (Stormacq et al., 2020).

PROmoting Successful Weight Loss in Primary CarE in Louisiana (PROPEL) trial aimed to develop and test the effectiveness of a 24-month, patient-centered, pragmatic health literacy directed obesity treatment program delivered within primary care in an underserved population. This current study reports on the association between health literacy and program effectiveness and examines potential correlates of weight loss related to patients' program adherence.

## Methods

As reported previously, PROPEL was a cluster-randomized, two-arm trial conducted in primary care clinics (Katzmarzyk et al., 2020). A total of 18 primary care clinics with predominately Black patients with low income from urban and rural areas across Louisiana were randomized to either a (1) high-intensity health lifestyle intervention group or (2) usual care group. All enrolled patients received the intervention to which their clinic was assigned. Outcomes were assessed at baseline and at the 6-, 12-, 18-, and 24-month visits (Katzmarzyk et al., 2020).

### Stakeholder and Patient Engagement

To help ensure cultural appropriateness, the design of all intervention materials was guided by extensive stakeholder and patient engagement. Stakeholders included Chief Executive Officers and Medical Directors of Federally Qualified Health Centers, in addition to PCPs in study clinics. We also had three Patient Advisory Boards located in North and South Louisiana and New Orleans, Louisiana, which were instrumental in designing and adapting the intervention sessions and materials.

### Participant Identification and Recruitment

English-speaking patients bonded to a participating clinic were identified through their PCPs, electronic medical records, and research staff visiting the clinics. Patients were recruited by emails sent though health portals, posters and brochures in clinic waiting areas, and interactions with PROPEL staff in the clinic (Katzmarzyk et al., 2018).

The main eligibility criteria included being age 20 to 75 years and body mass index (BMI) of 30 kg/m2 to 50 kg/m2. Exclusionary criteria included participation in a weight loss program, using weight loss medication, bariatric surgery within the last 2 years, or losing >10 pounds within the last 6 months (Katzmarzyk et al., 2018). Pennington Biomedical Research Center Institutional Review Board approved the study and all participants provided written informed consent.

### Usual Care Group

Patients in the usual care group received routine usual care from their primary care team throughout the 24-month period. At baseline, PCPs received a brochure and a presentation focused on current guidelines for weight management in primary care settings and CMS reimbursement. Patients received three newsletters per year on selected topics including the importance of reducing sedentary behavior, sleep hygiene, family coping skills, and smoking cessation (Katzmarzyk et al., 2021).

### The Intervention

Patients received a literacy directed high-intensity lifestyle intervention, which was modified from previous successful behavioral lifestyle regimens (Diabetes Prevention Program Research Group, 2002; Ratner, 2006; Rickman et al., 2011; Wadden et al., 2006) and was consistent with the 2013 American Heart Association, American College of Cardiology, The Obesity Society Guidelines (Jensen et al., 2014). The modified intervention was designed to be accessible and understandable to those with limited health literacy. All materials and approaches were adapted to be health-literacy and culturally appropriate through extensive consultation with our Patient Advisory Board and the team's health literacy experts (T. D. and C. A.). The intervention was delivered in weekly sessions with trained health coaches (16 face-to-face and 6 via phone) during the first 6 months and at least monthly sessions for the remaining 18 months, alternating between face-to-face and phone sessions, with a total of 42 sessions.

During the first 6 months, participants were given packaged portion-controlled food and meal replacement shakes; these were included to enhance access to healthy food and increase understanding of portion control. Session topics included using portion-controlled foods and meal replacements, increasing physical activity, self-monitoring, structured diets, healthy snacking, and dealing with stress. Health coaches worked with participants to develop and adhere to personalized action plans focused on eating, activity, and stress management.

### Health Coaches and Participant Materials

Trained health coaches with degrees in nutrition, physical activity, or behavioral medicine were recruited from local communities and embedded in the community clinics. The coaches were provided a day-and-a-half of face-to-face interactive training in health literacy principles and motivational interviewing techniques (U.S. Department of Health and Human Services, 2010; Weiss, 2007). The research team held weekly webinars with coaches and discussed challenges and successes in working with participants.

### Assessment

Weight was measured during clinic visits at baseline, 6, 12, 18, and 24 months. The full assessment battery was reported previously (Katzmarzyk et al., 2018). For this report, we included age, gender, race, and health literacy. The REALM [Rapid Estimate of Adult Literacy in Medicine] short form, a seven-item health literacy screening tool was used to assess health literacy (Arozullah et al., 2007). Raw scores can be converted to reading grade levels, which are a marker for health literacy; those scoring below 7 are considered to have limited health literacy (reading below a 9th grade level).

### Statistical Analysis

Participation compliance variables were summarized descriptively, both overall and according to health literacy categories (adequate and limited). The analysis aim was to investigate if percent change in weight in the intervention group was related to participation and adherence to the intervention, and whether the effectiveness of the intervention, relative to Usual Care, differed by health literacy.

We employed linear mixed models for repeated measures according to the intention-to-treat principle and assumed data were missing at random. All available percent weight changes at each follow-up visit were used as dependent variables with time-invariant participation compliance as the main independent variable. To allow for heterogeneity of intercepts across time, months (6, 12, 18, and 24 months) were included in the model, which further adjusted for race, age, gender, and the random effect of clinics. We further ran a model allowing each month to have different regression slopes with a test of the adherence variable-by-month interaction term. We report only the composite slope for clinically meaningful interpretation.

In addition, another model was created to inspect the differential effect of health literacy on the relationship. We used a similar repeated measures mixed model, having independent variables as compliance variable, month, subject-level covariates, health literacy and a health literacy-by-compliance interaction term. The regression coefficients for two health literacy categories as well as the significance test of between-category coefficients difference were obtained from the interaction term.

To discern whether there were significant differences in percent weight loss among patients with adequate and limited health literacy across intervention and usual care groups, we employed another repeated-measures linear mixed-effects multilevel model. This analysis considered percent weight loss as the dependent variable, with the group (intervention, usual care), assessment time, health literacy status, and their 3-way interactions as the independent variables. The model also included race, age, and gender to account for potential confounding factors. Additionally, a random cluster effect for clinics was incorporated into the model to address potential variability across different clinic settings. All analyses were conducted with SAS software, version 9.4.

## Results

Of the 803 patients enrolled in the trial, 84.4% were women, 67.4% were Black, 84.4% had an income <$40,000, and 31% had limited health literacy. The average BMI was 37.2 [standard deviation: 4.7] kg/m2. More patients (*n* = 452) were assigned to the intervention group than usual care (*n* = 351). There was a greater proportion of Black participants and women in the intervention group (**Table 1**). Overall, 16.6% of patients were lost to follow up (19.9% in the intervention group and 12.3% in the usual care arm). At 24 months, 18.3% of patients with adequate literacy and 12.6% with limited literacy were lost to follow up. We defined loss to follow-up as the percentage of patients who contributed baseline data but did not return for the 24-month assessment visit.

**Table 1:**
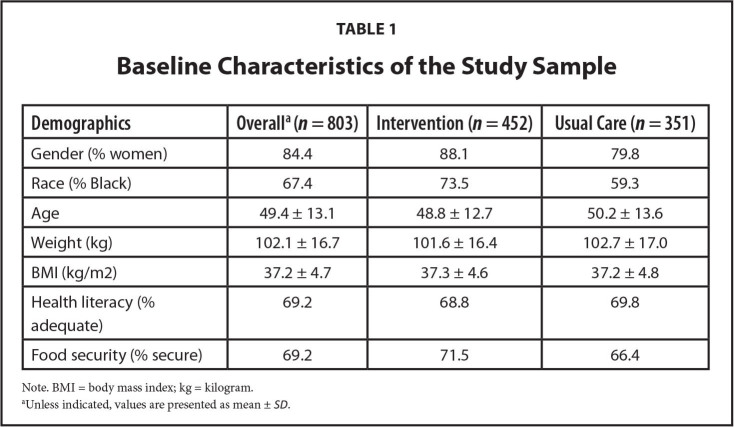
Baseline Characteristics of the Study Sample

**Demographics**	**Overall^a^(*n* = 803)**	**Intervention (*n* = 452)**	**Usual Care (*n* = 351)**
Gender (% women)	84.4	88.1	79.8
Race (% Black)	67.4	73.5	59.3
Age	49.4 ± 13.1	48.8 ± 12.7	50.2 ± 13.6
Weight (kg)	102.1 ± 16.7	101.6 ± 16.4	102.7 ± 17.0
BMI (kg/m2)	37.2 ± 4.7	37.3 ± 4.6	37.2 ± 4.8
Health literacy (%adequate)	69.2	68.8	69.8
Food security (% secure)	69.2	71.5	66.4

Note. BMI = body mass index; kg = kilogram.

a Unless indicated, values are presented as mean ± *SD*.

**Table 2** presents the participants characteristics and compliance, both overall and by health literacy level in the intervention group. Patients in the intervention group received 82.4% of coaching sessions (82.8% for adequate literacy versus 81.4% for limited literacy). Adjusted for age, race, and gender, there was a significant effect of total coaching sessions received, i.e., the greater the number of sessions received, the greater the percent weight loss (−0.230 ± 0.026 change in percentage weight change per lesson received, *p* < .0001) (**Table 3**). The benefit in weight loss was greater for patients with adequate health literacy (−0.280 ± 0.032, *p* < .0001), compared to patients with limited health literacy (−0.163 ± 0.045, *p* = .0004). These results are similar whether expressed as total materials received, percentage of materials received, or the number of distinct sessions attended. This effect persisted for those who received in-person sessions, while the number of remote sessions received was not related to weight loss.

**Table 2:**
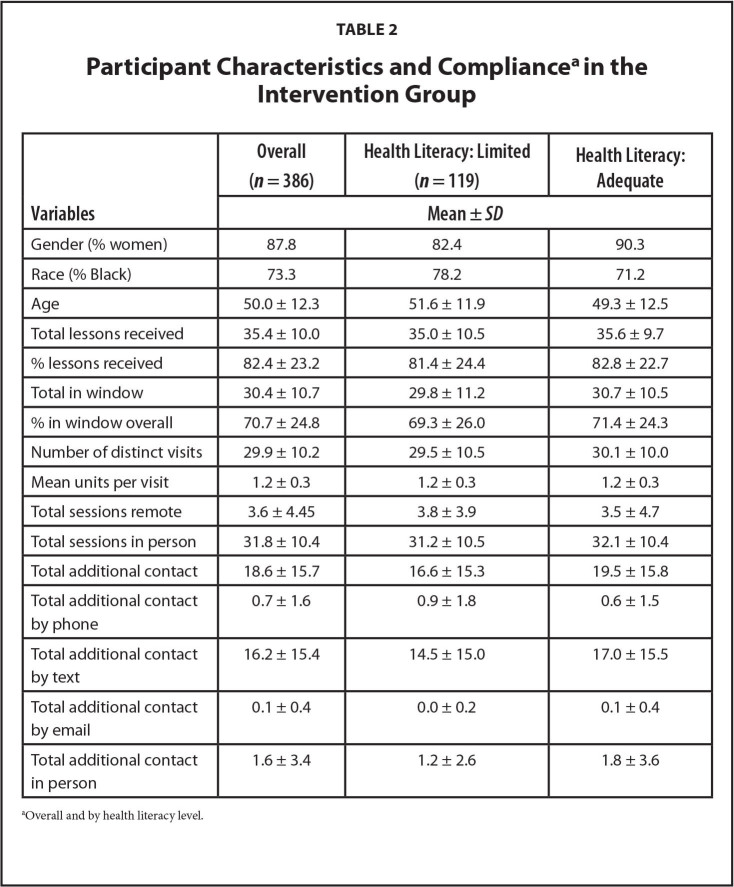
Participant Characteristics and Compliance^a^in the Intervention Group

**Variables**	**Overall (*n* = 386)**	**Health Literacy: Limited (*n* = 119)**	**Health Literacy: Adequate**
	**Mean ± *SD***		
Gender (% women)	87.8	82.4	90.3
Race (% Black)	73.3	78.2	71.2
Age	50.0 ± 12.3	51.6 ± 11.9	49.3 ± 12.5
Total lessons received	35.4 ± 10.0	35.0 ± 10.5	35.6 ± 9.7
% lessons received	82.4 ± 23.2	81.4 ± 24.4	82.8 ± 22.7
Total in window	30.4 ± 10.7	29.8 ± 11.2	30.7 ± 10.5
% in window overall	70.7 ± 24.8	69.3 ± 26.0	71.4 ± 24.3
Number of distinct visits	29.9 ± 10.2	29.5 ± 10.5	30.1 ± 10.0
Mean units per visit	1.2 ± 0.3	1.2 ± 0.3	1.2 ± 0.3
Total sessions remote	3.6 ± 4.45	3.8 ± 3.9	3.5 ± 4.7
Total sessions in person	31.8 ± 10.4	31.2 ± 10.5	32.1 ± 10.4
Total additional contact	18.6 ± 15.7	16.6 ± 15.3	19.5 ± 15.8
Total additional contact by phone	0.7 ± 1.6	0.9 ± 1.8	0.6 ± 1.5
Total additional contact by text	16.2 ± 15.4	14.5 ± 15.0	17.0 ± 15.5
Total additional contact by email	0.1 ± 0.4	0.0 ± 0.2	0.1 ± 0.4
Total additional contact in person	1.6 ± 3.4	1.2 ± 2.6	1.8 ± 3.6

a Overall and by health literacy level.

**Table 3:**
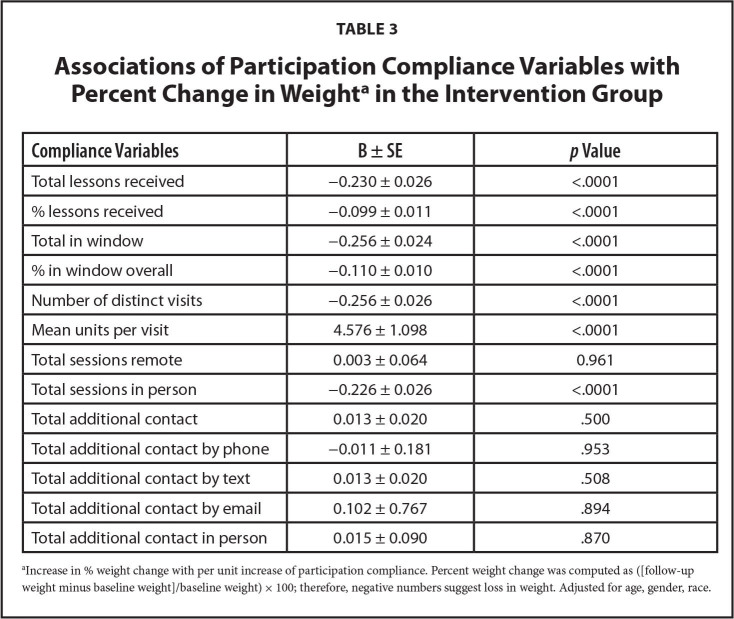
Associations of Participation Compliance Variables with Percent Change in Weight^a^ in the Intervention Group

**Compliance Variables**	**Β ± SE**	***p* Value**
Total lessons received	−0.230 ± 0.026	<.0001
% lessons received	−0.099 ± 0.011	<.0001
Total in window	−0.256 ± 0.024	<.0001
% in window overall	−0.110 ± 0.010	<.0001
Number of distinct visits	−0.256 ± 0.026	<.0001
Mean units per visit	4.576 ± 1.098	<.0001
Total sessions remote	0.003 ± 0.064	0.961
Total sessions in person	−0.226 ± 0.026	<.0001
Total additional contact	0.013 ± 0.020	.500
Total additional contact by phone	−0.011 ± 0.181	.953
Total additional contact by text	0.013 ± 0.020	.508
Total additional contact by email	0.102 ± 0.767	.894
Total additional contact in person	0.015 ± 0.090	.870

a Increase in % weight change with per unit increase of participation compliance. Percent weight change was computed as ([follow-up weight minus baseline weight]/baseline weight) × 100; therefore, negative numbers suggest loss in weight. Adjusted for age, gender, race.

The greater the number of lessons covered in a single session was found to be detrimental to weight loss (4.576 ± 1.098 change in percentage weight change with one more unit covered per session, *p* < .0001). Additional contacts, whether it be phone, text, email, or in-person, had no effect on weight loss (**Table 3**).

**Figure 1.**
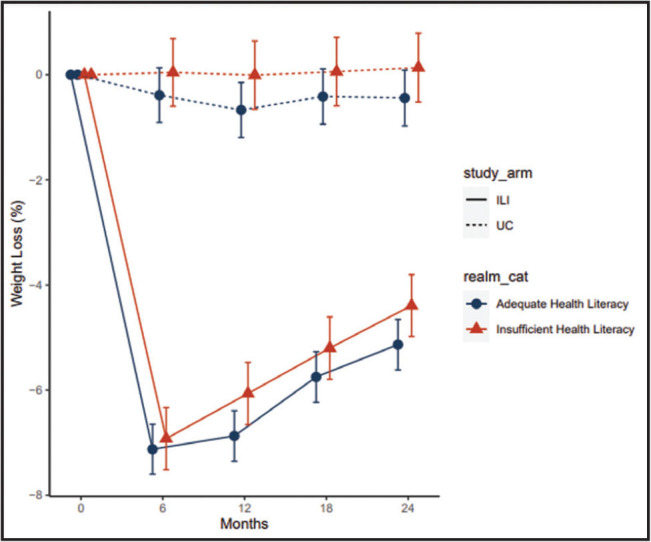
Percent weight loss of PROPEL (PROmoting Successful Weight Loss in Primary CarE in Louisiana) patients for more than 2 years by health literacy status and Usual Care (UC) versus the health literacy directed lifestyle intervention (HLI) group.

**Figure 1** shows percent weight change by health literacy status and study group at each month. In the usual care group, patients lost an average of 0.44% of their enrollment weight after 24 months. Those with adequate literacy lost 0.57% and those with low literacy 0.30%, which was not significantly different. In the intervention group, patients lost an average of 4.9% of their enrollment weight at 24 months. Those with adequate literacy lost 5.2% and those with low literacy 4.7%, which was not significantly different.

The association between health literacy and intervention effectiveness was consistent across the 24-month study period, though not statistically significant. In the intervention group, patients with adequate health literacy had greater weight loss by a margin of 0.50 ± 0.75 (*p* = .50), while in usual care group, the margin was 0.27 ± 0.84 (*p* = .74). The percent weight loss difference between intervention and usual care groups was 4.6 ± 0.8 (*p* < .001) among patients with adequate health literacy and 4.4 ± 1.0 (*p* < .001) among patients with limited health literacy. The difference in percent weight loss between the intervention and usual care groups was 0.2 ± 1.1 (*p* = .84) higher for adequate literacy patients.

## Discussion

Weight loss in both arms of the trial were similar among those with low and adequate health literacy. Further, the effectiveness of the intervention, relative to usual care, did not differ by level of health literacy. However, the greater the number of in-person health coaching sessions a patient received, the greater the weight loss, suggesting that exposure to the intervention materials and interactions with the health coach are important determinants of weight loss success. Our clinic-based study is consistent with others that found weight loss strategies in primary care clinics have limited success without longer term interventions designed to improve patients' knowledge and skills for weight loss (Ahern et al., 2017; de Lannoy et al., 2021; Faruqi et al., 2015; Katzmarzyk et al., 2021; Steinberg et al., 2019).

Primary care-based weight loss interventions reported in the literature varied in goals (weight loss, preventing weight gain, improving diet quality) duration, intensity (number of contacts with a health professional), mode of delivery (in-person, phone call, text digital), and patient demographics, BMI ranges, and clinics in countries with differing health systems (Ahern et al., 2017; de Lannoy et al., 2021; Faruqi et al., 2015; Katzmarzyk et al., 2021; Lanpher et al., 2016; Semlitsch et al., 2019; Steinberg et al., 2019). The most effective interventions lasted 12–24 months; however, all found sustaining weight loss long term was challenging (Ahern et al., 2017; Katzmarzyk et al., 2021; McVay et al., 2019).

Our intervention is consistent with findings of a systematic review of health literacy interventions in primary care clinics, which found patients with obesity need strategies involving continuing lifestyle change, regular monitoring and support of trained health professionals (Faruqi et al., 2015). Although not specific to health literacy, Steinberg et al. (2019) also found weekly self-monitoring, as well as tailored feedback on diet and exercise from a dietician or provider counselor was beneficial for vulnerable primary care patients. Lanpher et al. (2016) found that it was a major challenge to design obesity treatments that are accessible to patients across the spectrum of health literacy. She reported concrete instructions on how to change behavior, progress reports that track changes in behavior, and written health materials are effective in producing weight loss. Our study, like these previous studies in primary care settings, was multicomponent intervention targeting nutrition, physical activity and ongoing interactive personal coaching.

A health literacy designed digital obesity prevention intervention among Black women (55% low health literacy) in North Carolina community clinics (Lanpher et al., 2016) found no significant difference in sessions attended or change in weight by literacy. Our study had similar findings. In both Lanpher's 2016 study and the present study, low health literacy did not affect attendance or weight loss. In the present study, those with higher health literacy benefited more from receiving literacy-appropriate sessions and materials. At 24 months, the effect of the number of sessions was still present, but the interactions with health literacy were not. However, our study found the greater number of in-person interactive health coaching sessions a patient received, the greater the weight loss, but the number of remote sessions received was not related to weight loss. Coaches reported patients sometimes responded to these messages/calls while busy or distracted at work or at the grocery store or with children. However, we have no objective data from patients and found no mention in the literature of this communication problem.

In our study, giving patients additional material in a session to make up for lost sessions was not beneficial or effective in promoting weight loss. This is noteworthy, although we found no reports of this phenomenon in the literature. It seems cramming material from more than one session into a single session to make up for missed sessions was not beneficial; perhaps this leads to information overload. In our study, face-to-face sessions were most effective, additional contacts by phone, text, email, or in-person, had negligible effects on weight loss. Perhaps regular personal relationships promoted patient trust, engagement, communication, and accountability.

This intervention of predominately low-income participants designed to be culturally and literacy appropriate mitigated the impact of low health literacy. There were no significant differences in intervention effectiveness among patients with low or adequate health literacy, suggesting that our adaptations to the program materials were successful.

## Study Limitations

This study was limited to English-speaking primary care and community clinic patients in Louisiana. The predominance of women and Black adults prevented gender- and race-specific analyses. The association between the number of sessions attended and weight loss is observational and needs to be confirmed using randomized study designs. The degree to which this observation is due to reverse causation or caused by factors not measured in this study is not known. The lost-to-follow-up rates differed somewhat between the two study arms (19.9% in the intervention group and 12.3% in the usual care group). The degree to which this difference introduced bias into the results is not known.

## Conclusion

This community clinic-based literacy-directed obesity treatment program delivered by embedded health coaches was effective in engaging low-income, underserved patients and helping them lose weight. The health literacy directed intervention was designed to be accessible, understandable culturally appropriate, and useful for patients with limited literacy and income. This mediated the impact of low health literacy. Future studies may need to consider the potential positive impact of ongoing personal relationships on patient engagement. Interventions with vulnerable populations also need to consider the multiple challenges disadvantaged populations encounter in their daily life and its impact on weight loss. They also need to work with the disadvantaged patients and their providers in designing feasible strategies to address barriers to patients' ability to lose weight and maintain weight.
